# Tocotrienol Supplementation Improves Late-Phase Fracture Healing Compared to Alpha-Tocopherol in a Rat Model of Postmenopausal Osteoporosis: A Biomechanical Evaluation

**DOI:** 10.1155/2012/372878

**Published:** 2012-07-05

**Authors:** Sharlina Mohamad, Ahmad Nazrun Shuid, Sabarul Afian Mokhtar, Shahrum Abdullah, Ima Nirwana Soelaiman

**Affiliations:** ^1^Department of Pharmacology, Faculty of Medicine, Universiti Kebangsaan Malaysia, Jalan Raja Muda Abd Aziz, 50300 Kuala Lumpur, Malaysia; ^2^Cluster of Integrative Medicine, Advanced Medical and Dental Institute (AMDI), Universiti Sains Malaysia, 13200 Kepala Batas, Pulau Pinang, Malaysia; ^3^Department of Orthopedics, UKM Medical Centre, Jalan Yaacob Latiff, Bandar Tun Razak, Cheras, 56000 Kuala Lumpur, Malaysia; ^4^Department of Mechanical and Materials Engineering, Faculty of Engineering, Universiti Kebangsaan Malaysia, 43600 Bangi, Selangor, Malaysia

## Abstract

This study investigated the effects of **α**-tocopherol and palm oil tocotrienol supplementations on bone fracture healing in postmenopausal osteoporosis rats. 32 female Sprague-Dawley rats were divided into four groups. The first group was sham operated (SO), while the others were ovariectomised. After 2 months, the right femora were fractured under anesthesia and fixed with K-wire. The SO and ovariectomised-control rats (OVXC) were given olive oil (vehicle), while both the alpha-tocopherol (ATF) and tocotrienol-enriched fraction (TEF) groups were given alpha-tocopherol and tocotrienol-enriched fraction, respectively, at the dose of 60 mg/kg via oral gavages 6 days per week for 8 weeks. The rats were then euthanized and the femora dissected out for bone biomechanical testing to assess their strength. The callous of the TEF group had significantly higher stress parameter than the SO and OVXC groups. Only the SO group showed significantly higher strain parameter compared to the other treatment groups. The load parameter of the OVXC and ATF groups was significantly lower than the SO group. There was no significant difference in the Young's modulus between the groups. In conclusion, tocotrienol is better than **α**-tocopherol in improving the biomechanical properties of the fracture callous in postmenopausal osteoporosis rat model.

## 1. Introduction

Osteoporosis is a disease characterized by low bone mass and microarchitectural deterioration of bony tissue, which leads to bone fragility and an increase in fracture risk [1]. Osteoporosis has affected more than 75% of the population in Europe, US, and Japan [2] and is estimated to cause more than 50% of hip fractures in Asia by the year 2050 [3, 4]. Osteoporosis causes bone loss, leading to fragile bone which fractures under slight trauma. Apart from increasing the incidence of atraumatic or osteoporotic fractures, it may also add severity to a traumatic fracture and delayed fracture healing. Several animal studies have confirmed that osteoporotic bones undergo prolonged and impaired healing process [5, 6]. The incidence of osteoporotic fractures is expected to be on the rise, with the growing number of elderly people worldwide [7, 8].

Fracture healing is a complex process with three distinct phases, that is, the reactive phase, reparative phase, and the remodeling phase [9]. In the reactive phase, inflammation and formation of granulation tissue occur immediately after fracture. This is followed by the reparative phase, where callus is formed and lamellar bone is deposited. In the final or remodeling phase, the bone will be remodeled back to its original bone contour. Studies have shown that osteoporosis can delay and impair fracture healing [10]. Antiosteoporotic drugs such as estrogens, vitamin D, and bisphosphonates may also influence fracture healing. 

Due to these complexities and ethical issues, animal osteoporotic models are more appropriate to study the effects of osteoporosis on the fracture repair process [11]. These animal models include small animals such as rat and mouse to larger animals such as dogs and sheep. Ovariectomised rat has been widely used and accepted as osteopenia model to simulate bone loss in postmenopausal women [12, 13]. There are many similar bone changes in postovariectomised rats and postmenopausal women, making the ovariectomised rat a suitable model for postmenopausal bone loss [14]. The same model can be used to study fracture healing in postmenopausal osteoporosis.

The National Institutes of Health consensus panel defined that bone quality is an important parameter for the evaluation of osteoporosis, fracture risk, and osteoporosis treatment. The panel also suggested that the bone biomechanical strength is compromised with low bone density and poor bone quality [15]. The mechanical properties of bone can be assessed at different levels from the macroscopic to the ultramacroscopic levels and under different mechanical basic assumptions, such as heterogenous or homogenous and isotropic or anisotropic assumptions [16]. Bone mineral density (BMD) measurement is used widely for the detection of osteoporosis and is the major determinant of the biomechanical functioning of an osteoporotic bone. Dual energy X-ray absorptiometry (DEXA) is a good noninvasive method for BMD assessment [17].

Bone structure is made up of cortical bone (outer layer) and trabecular bone (inner layer). The inner layer has a spongy and honeycomb-like structure which is essential for the bone strength. The strength of a bone is normally estimated indirectly from its density, whereby a high density bone is assumed to be strong. This assumption may not always be accurate as discovered with the fluoride treatment of osteoporosis. The bone density appeared to be improved with fluoride treatment but in actual fact there was no improvement in strength as the new bones formed were fragile [18–21].

The bone strength can be measured directly with biomechanical testing technique, where the strength (stress), elasticity of the bone (strain), energy needed before the bone deform (load), and elastic modulus (Young's modulus) were evaluated. Biomechanical testing provides an accurate measurement of bone strength but can only be carried out in an animal model as the bone samples need to be subjected to force until they break. This is another reason why animal model is appropriate for fracture healing studies.

The strength of bone is determined by its material composition and structure [22]. Biomechanical testing of healing fractures is a useful tool in evaluating fracture healing. Bone stiffness is important to ensure that the bones will not bend when loaded, but at the same time they must also be flexible to absorb the energy imposed by the loading via elastic and plastic deformation [23].

Bending tests was proven to be sensitive in measurements of the mechanical properties of healing callus in rat [24]. In the case of fracture healing, the biomechanical testing can be carried out on the callous, the hard bony tissue that develops around the ends of a fractured bone during healing.

Vitamin E is a lipid soluble vitamin which has a chain breaking ability to stop lipid peroxidation chain reaction. Oxidative stress has been linked to the pathogenesis of bone loss leading to osteoporosis [25]. Therefore, an antioxidant such as vitamin E is able to protect bone against osteoporosis via its antioxidant properties. There are 2 types of vitamin E, tocopherol and tocotrienol, which are further divided into 4 isomers, *α*, *β*, *γ*, and *δ*. Tocotrienol is a unique vitamin E which is abundant in palm oil from *E. guinesses *species. It has better antioxidant capacity than tocopherol [26, 27]. Recently, tocotrienol has become a research interest not only for its potent antioxidant activity but also for its anticholesterol [28], antiplatelet [29], and anticancer properties [30, 31]. A study by Norazlina et al. found that both palm vitamin E and *α*-tocopherol maintained bone mineral density in ovariectomised rats [32]. However, there are still limited numbers of studies focusing on the effects of vitamin E on bone, especially tocotrienol. Durak et al. reported that *α*-tocopherol had positive effect on the fracture healing of a rabbit model [33]. Similar findings on *α*-tocopherol were seen in another study by Keskin et al. [34].

The present study was designed to investigate the effects of tocotrienol supplementation on bone fracture healing in postmenopausal osteoporosis rats in comparison to *α*-tocopherol supplementation.

## 2. Materials and Methods

### 2.1. Animals and Treatment

**Figure 1:**
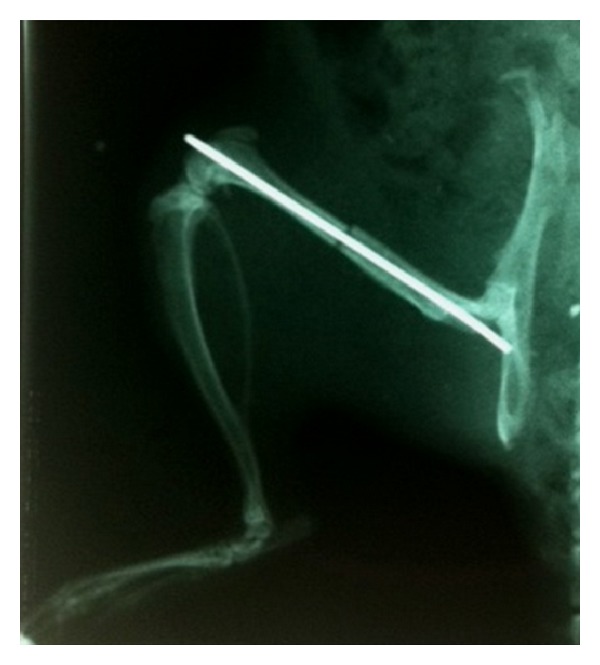
X-ray image taken for confirmation of K-wire insertion and the fracture is at the mid-diaphyseal region.

32 female Sprague-Dawley rats weighing between 250 to 300 grams were used in this study. They were obtained from the Laboratory Animal Resources Unit, Faculty of Medicine, Universiti Kebangsaan Malaysia. The rats were divided into four groups, where the first group was sham operated (SO), while the other three groups were ovariectomised. They were left untreated for two months to allow for osteoporosis to develop in the ovariectomised rats. The right femora of all the rats were then fractured using a blunt guillotine method according to Vialle et al. [35] under anesthesia, and the procedures were done aseptically. Ketamine and Xylazil (1 : 1 ratio) were given intramuscularly at the dose of 0.1 mL/100 g weight as anesthetic. Iodine was then applied and a small incision was made at the right knee. The right patella was then dislocated and a Kirshner wire (K-wire) (1.0 mm in diameter) was inserted with a drill into the intramedullary canal of the femur as the internal fixation to the bone. The patella was then relocated back and the incision was sutured using a nonabsorbable suture (nylon 4). A fracturer device was used to fracture the right femur of all the rats. This device uses the same principle as the guillotine principle, where a 500 g steel blunt bar was dropped on the mid diaphysis of the femur supported by two metal bases. This forms a three-point system, which could produce a closed and standardized fracture. This device measuring 12.5 cm by 45 cm has three platforms, with the middle platform is 30 cm height from the lowest platform and 10 cm height to the upper platform. Triggering bar is placed at the upper platform to release the bar. The upper platform also functions to stabilize the whole structure of the device. Baytril was given intramuscularly for perioperative antibiotic prophylaxis. Buprenorphine (analgesics) was injected subcutaneously to the rats every 12 hours at the dose of 0.3 mg/kg rat weight. For confirmation purpose, X-rays were taken immediately after fracture using X-ray machine (Proteus XR/a, GE UK). This is to confirm that fractures had occurred at mid diaphysis of the right femur and the K-wires were inserted correctly (Figure 1). The rats were allowed unrestricted weight bearing after recovery from anesthesia.

The ovariectomised rats were then randomly divided into three groups, which are ovariectomised control (OVXC), *α*-tocopherol (ATF), and tocotrienol-enriched fraction (TEF) groups. All the rats were housed individually in separate cages at room temperature under natural day/night cycle (12 hours light/dark cycle). All the rats were given rat chow (Gold Coin, Malaysia), and deionized water was given *ad libitum*. The ATF-treated group was given *α*-tocopherol acetate (Sigma, USA), whereas the TEF group was given Gold-Tri E (Golden Hope Bioganic Sdn. Bhd., Malaysia), both at the dose of 60 mg/kg rat weight. The Gold-Tri E (Batch no. GHB071113196298R) was composed of 73.9 mg/g *α*-tocopherol, 167.1 mg/g *α*-tocotrienol, 41.1 mg/g *β*-tocotrienol, 165.2 mg/g *γ*-tocotrienol, and 98.5 mg/g *δ*-tocotrienol. On the other hand, SO and OVXC groups were given olive oil (Bertolli, Italy) which acts as vehicle. Olive oil was used as vehicle because it only contains a very small amount of *α*-tocopherols (51 p.p.m) and no tocotrienols. All the treatments were given via oral gavages for 2 months [36], 6 days per week. After 2 months, all the rats were euthanized and their femora were dissected out. This study has been approved by the UKM Animal Ethics Committee (FP/FAR/2008/NAZRUN/13-FEB/217-FEB2008-FEB2010).

### 2.2. Bone Biomechanical Test

**Figure 2:**
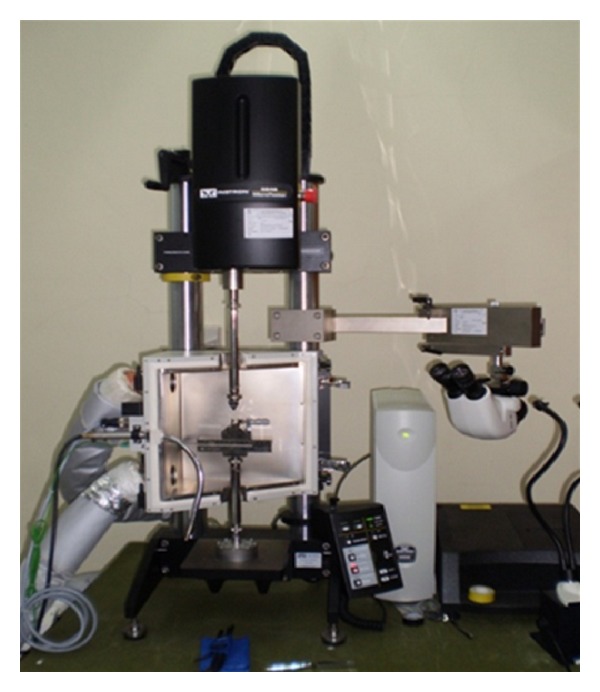
Instron machine with Bluehill software that we used to assess the bone strength.

The right femora samples were prepared for bone biomechanical test. They were wrapped individually in gauze dipped in phosphate buffer saline (PBS) to make sure that the bones did not dry out and were kept in 4°C temperature to avoid bone degradation. The biomechanical strength of the healed bones was assessed using Instron machine (Instron Microtester 5848, Instron Corp, USA) with Bluehill software (Figure 2). This machine employs the 3-point bending test, where the load was applied to the callous at the mid-point of the femoral diaphysis at the speed of 5 mm/min until it refractured. The stress, strain, and load parameters were recorded by the software. From the stress versus strain graph plotted, Young's modulus was derived from the curve gradient.

### 2.3. Statistical Analysis

Results were expressed as mean ± standard error mean (SEM). Analysis was done using Statistical Package for Social Sciences software (SPSS version 19.0, USA). Normality of the data was tested using Kolmogorov-Smirnov test. ANOVA was performed for normally distributed data, followed by Tukey's HSD. The level of significance was taken as *P* < 0.05.

## 3. Results

**Figure 3:**
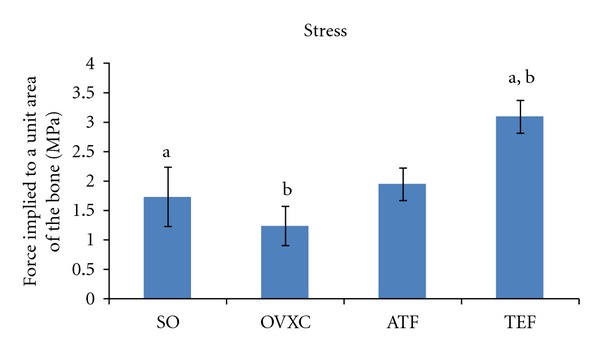
Bar chart shows stress parameter derived from the bone biomechanical testing. SO: sham-operated group. OVXC: ovariectomised control group. ATF: ovariectomised + ATF supplemented group. TEF: ovariectomised + TEF supplemented group. Values marked with the same alphabet are significantly different at *P* < 0.05.

The strength of the healed bone (callous) was assessed using the biomechanical testing. Stress is the force implied to a unit area. Higher stress parameter indicated higher callous strength, as more force is needed to flex the callous. The callous of the TEF group had significantly higher stress parameter than the SO and OVXC groups. This indicates that the fractured femora of the TEF group had better healing than both these groups. There was no significant difference in the stress parameter between the TEF and ATF groups (Figure 3).

**Figure 4:**
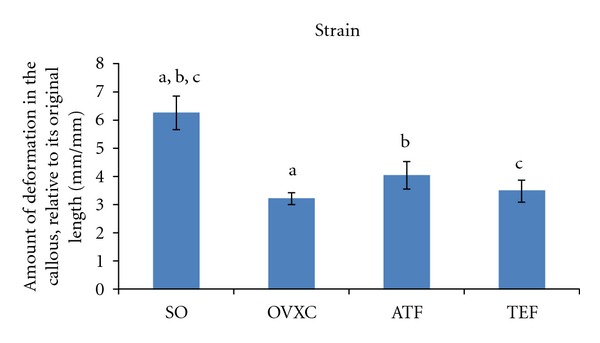
Bar chart shows strain parameter derived from the bone biomechanical testing. SO: sham-operated group. OVXC: ovariectomised control group. ATF: ovariectomised + ATF supplemented group. TEF: ovariectomised + TEF supplemented group. Values marked with the same alphabet are significantly different at *P* < 0.05.

Strain represents the amount of deformation in the callous, relative to its original length. Only the SO group showed significantly higher strain parameter compared to the other treatment groups. There was no other significant finding in the strain parameter between the other groups (Figure 4).

**Figure 5:**
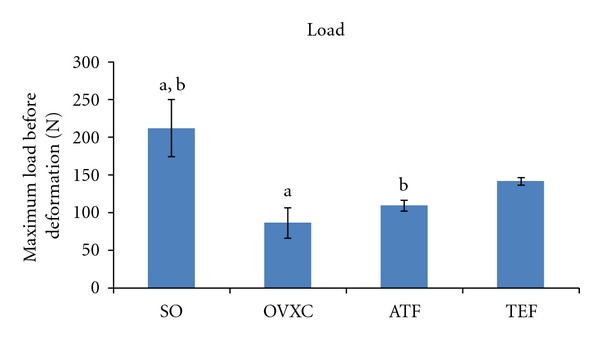
Bar chart shows load parameter derived from the bone biomechanical testing. SO: sham-operated group. OVXC: ovariectomised control group. ATF: ovariectomised + ATF supplemented group. TEF: ovariectomised + TEF supplemented group. Values marked with the same alphabet are significantly different at *P* < 0.05.

Load represents the maximum load that the callous is able to receive before it undergoes deformation. The Load parameter of the OVXC and ATF groups was significantly lower than the SO group. There was no significant difference in the Load parameter between the SO and TEF groups (Figure 5).

**Figure 6:**
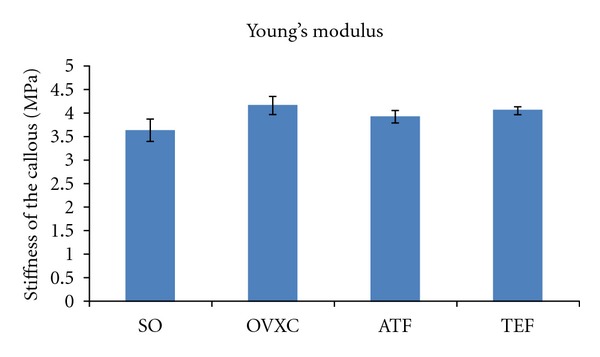
Bar chart shows Young's modulus derived from the bone biomechanical testing. SO: sham-operated group. OVXC: ovariectomised control group. ATF: ovariectomised + ATF supplemented group. TEF: ovariectomised + TEF supplemented group. Values marked with the same alphabet are significantly different at *P* < 0.05.

Young's modulus represents the stiffness of the callous. It is derived from the slope of the elastic region of the stress-strain curve. There was no significant difference in the Young's modulus between the groups (Figure 6).

## 4. Discussions

Osteoporotic or pathological fracture is the most common complication of osteoporosis which occurs with minimal trauma due to bone fragility. In the present study, comparison was made between the effects of ATF and TEF supplementations on the fracture healing of postmenopausal osteoporosis rat model. The healing was assessed by measuring the strength of the callous at the fracture site with biomechanical testing. The callous biomechanical properties of the treatment groups were compared to the SO and OVXC groups. The latter two groups acted as the control groups and represented the models for traumatic fracture and osteoporotic fracture, respectively.

In terms of safety, the 60 mg/kg dosage of tocotrienol-enriched fraction and *α*-tocopherol used in the present study were nontoxic to the rats. Toxicity studies in rat models showed NOAEL (no observable adverse effect level) with the tocotrienol dose of 130 mg/kg [37] to 2500 mg/kg weight [38]. While for *α*-tocopherol, the NOAEL level was found to be at 643 mg/kg [39].

Osteoporosis has been shown to impair bone fracture healing. It influences both the early and late period of fracture healing in rat osteoporotic model [22, 23, 36]. The callous strength is an important indicator to show that the fracture healing is complete with full restoration of its structure and function. Calcium supplementation was reported to promote fracture healing in ovariectomised rats based on radiological assessments. However, biomechanical testing confirmed that the fracture healing was not accompanied by improvement in the callous strength [40]. Therefore, despite of the reports that vitamin E was able to reverse osteoporosis, it is important to determine whether vitamin E would improve the callous strength during fracture healing of osteoporotic bone.

Most bone studies have found that tocotrienols were better than tocopherols in preventing osteoporosis (Nazrun et al., 2010) [41], while Norazlina et al. found that palm vitamin E (rich in tocotrienol) was comparable to *α*-tocopherol in maintaining the bone mineral density of ovariectomised rats [32].

Our findings showed that the bone fractures of the ovariectomised rats healed poorly as demonstrated by the lower strain and load parameters of their calluses. Supplementation with *α*-tocopherol failed to improve any of the biomechanical parameters of the callous and therefore did not seem to promote the fracture healing of ovariectomised rats. Supplementation with TEF was able to significantly increase the stress and load parameters compared to OVXC group. It is also interesting to discover that the stress parameter was also significantly higher than the sham-operated group. This indicated that tocotrienol was not only able to promote fracture healing but may further strengthen the fracture callous. This is consistent with the findings by Shuid et al., which showed that normal male rats supplemented with *γ*-tocotrienol had better bone biomechanical strength than the normal control rats [41].

Turk et al. reported that *α*-tocopherol has a positive effect on fracture healing [42]. The radiological evaluation showed that *α*-tocopherol supplementation had improved the fracture healing of normal rats. In another study, based on the radiological scoring of fracture healing, ovariectomised rats supplemented with *α*-tocopherol were found to have similar fracture healing to that of the sham-operated rats (Nazrun et al., 2011) [43]. In the present study, *α*-tocopherol supplementation failed to improve the callous biomechanical parameters of ovariectomised rats. This meant that even though *α*-tocopherol seems to promote fracture healing on radiological assessments, there was no improvement in the fracture callous strength. This is in line with Arjmandi et al. [44] who found that *α*-tocopherol has no effects on the bone biomechanical properties of rats.

The superiority of tocotrienol may be accounted by its better antioxidant capacity than *α*-tocopherol. Free radicals have been shown to be involved in the process of fracture healing and their excessive levels may impair fracture healing [45, 46]. Osteoporosis itself may worsen oxidative stress as seen in postmenopausal osteoporotic subjects, who were found to be under oxidative stress [47, 48]. It is believed that tocotrienol was able to overcome oxidative stress at the fracture site to create an ideal environment for fracture healing to take place [26].

In conclusion, supplementation with tocotrienol has improved the biomechanical properties of the fracture callous in postmenopausal osteoporosis rat model. Supplementation with *α*-tocopherol failed to produce similar effects. Further studies are required to explore the potential of tocotrienol in promoting fracture healing of osteoporotic bone.
